# Genome annotation of a cluster FD *Arthrobacter* bacteriophage Casab isolated from soil in Tennessee

**DOI:** 10.17912/micropub.biology.002353

**Published:** 2026-10-07

**Authors:** Sergei Markov, Samantha Bradford, Debra Bradley, Andrew Canlas, Haley Clendenning, Traonna Cunningham, Alexander Eberly, Abasiakan Godwin, Taylon Huycke, Micah Kubr, Emmalee Langlois, McKenzie Meyer, Cedric Myers, Neel Patel, Emaleigh Roland, Cassandra South, Tahmar Upshaw, Abigail Vela, Kylie Yun

**Affiliations:** 1 Biology, Austin Peay State University, Clarksville, TN, United States

## Abstract

Bacteriophage Casab was isolated from Tennessee garden bed soil sample using the bacterium *Arthrobacter globiformis* B-2979 *.* It has a 20274 bp genome with 29 predicted protein-coding genes, of which 19 were assigned predicted functions. Casab has a podovirus morphology and is grouped with actinobacteriophages in cluster FD. Casab encodes a glycoside hydrolase that is, notably, not present in other known FD cluster bacteriophages but can be found in a cluster AY bacteriophage.

**Figure 1. Transmission electron microscopy image of bacteriophage Casab. A short tail of 16-18 nm indicates podovirus morphology. The capsid diameter was 52-54 nm (n=4):** Samples for electron microscopy were stained using 1% uranyl acetate on grids attached to Pelco Tabs (Ted Peller, Inc., Redding, CA). The photo was taken using a Hitachi H-7650 Transmission Electron Microscope (Tokyo, Japan) with an accelerating voltage of 100 kV.

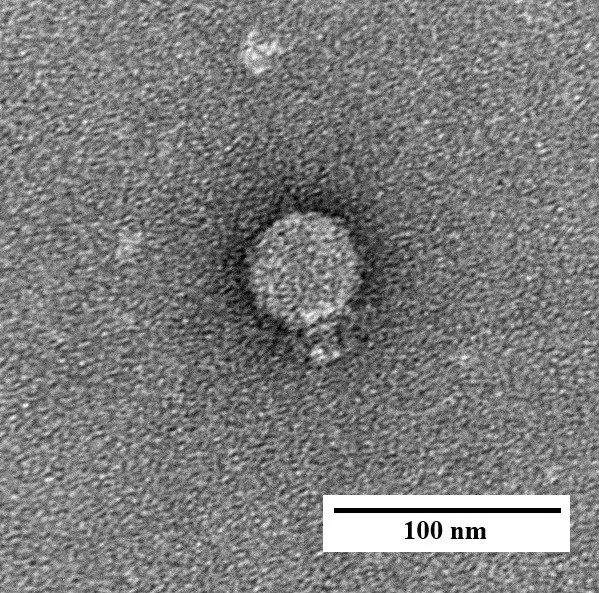



## Description

Bacteriophages have an essential role in the environment as bacterial population regulators and were crucial in advancing biotechnology and medicine (Hatfull, 2020). Here, we describe the isolation, DNA sequencing and genome annotation of bacteriophage Casab.

The bacteriophage was isolated from a garden bed soil sample near Clarksville, Tennessee (GPS coordinates 36.47717 N, 87.19055 W). The bacterium *Arthrobacter globiformis* B-2979 was used as the host for isolating Casab. Standard enriched isolation methods were followed for isolation (Zorawik et al., 2024; Markov et al. 2026). The sample was collected at 17°C and then incubated in peptone-yeast calcium (PYCa) liquid medium for 2 hours at 30°C. After incubation, the sample was centrifuged at 2,000 g for 10 minutes. The supernatant was passed through a 0.22- µm-pore filter, and the filtrate was inoculated with *A. globiformis* and incubated with shaking at 250 rpm for 2 days at 30°C. After incubation, the sample was centrifuged at 2,000 g for 1 minute to pellet the bacteria. The supernatant was then filtered and the filtrate (10 µL) added to PYCa top agar with *A. globiformis* and incubated for 2 days at 30°C. Afterward, a plaque was selected and purified through two additional rounds of plating, yielding bacteriophage Casab which formed clear, round, medium-size plaques of 2-3 mm in diameter (n=4). Transmission electron microscopy of Casab showed that it has podovirus morphology with icosahedral capsid of 52-54 nm (n=4) and a short tail of 11-13 nm (n=4) (Fig. 1).

The genomic DNA was isolated from a lysate of Casab using the Wizard DNA Clean-Up Kit (Promega, Madison, WI). DNA was sequenced using Illumina NextSeq 1000 technology with an XLEAP-P1 Kit to yield 100-base single-end reads with 118-fold coverage (660,884 reads). DNA was enzymatically sheared for sequencing using the Ultra II Library Kit (NEB, Ipswich, MA). Trimming of raw reads was achieved with cutadapt 4.7 (using the option: –nextseq-trim 30), filtered by using skewer 0.2.2 (using the options: -q 20 -Q 30 -n -l 50), and assembled using Newbler v2.9 (Russell, 2018). The Consed v29 (Gordon et al., 1998) was used to check raw reads for genomic termini and completeness as previously described by Russell (2018). Casab has a 20274 bp genome and a GC content of 59.3%. FD cluster phages are predicted to have covalently attached proteins at the 5' termini.

Glimmer v3.02 (Delcher et al., 1999) and GeneMark v2.5p (Besemer and Borodovsky 2005) were used to predict Casab's genes. The genome was then manually annotated using Starterator v485.0 ( http://phages.wustl.edu/starterator ), Phamerator v2.5 (Cresawn et al., 2011), PECAAN v20211202.0 ( https://blog.kbrinsgd.org/ ), BLASTp (Altschul et al., 1990) against the NCBI non-redundant Actinobacteriophage databases, HHpred v3.2 (Söding et al., 2005) against the PDB_mmCIF70, Pfam v.37.0, and NCBI Conserved Domains databases v3.19. Default parameters were used for all programs. By this annotation process, 29 protein-coding genes were predicted in Casab, of which plausible functions were allocated for only 19 genes. No tRNA genes were found in Casab's genome by ARAGORN v1.2.41 (Laslett et al., 2004) and tRNAscan-SE v2.0 (Lowe, 2016). Casab was placed in cluster FD by using the Gene Content Similarity (GCS) tool of at least 35% to bacteriophages in the Actinobacteriophage database (Gauthier and Hatfull 2023). Currently, the cluster FD contains only nine bacteriophages. Casab is most closely related to the bacteriophage Dunamis (74.27% GCS only) isolated from soil in River Falls, WI.

Like other cluster FD phages, all but a small number of genes at the left end of the genome (5 genes in Casab) are reverse transcribed. At the other end of the genome, Casab notably encodes a predicted glycoside hydrolase (gp25) for which there is only a single homologue in the actinobacteriophage database, encoded by cluster AY phage Gorphy that was also isolated using *A. globiformis* B-2979.

**Data availability**

GenBank accession number for Casab is PZ492774 and SRA accession number is SRR37265634 .
